# Reply to the ‘Comment on “A quantitative definition of hypervalency”’ by R. D. Harcourt and T. M. Klapötke, *Chem. Sci.*, 2016, **7**, DOI: ; 10.1039/C5SC04866D

**DOI:** 10.1039/c6sc00859c

**Published:** 2016-02-25

**Authors:** Marcus C. Durrant

**Affiliations:** a Department of Applied Sciences , Northumbria University , Newcastle-upon-Tyne , NE1 8ST , UK . Email: marcus.durrant@northumbria.ac.uk

## Abstract

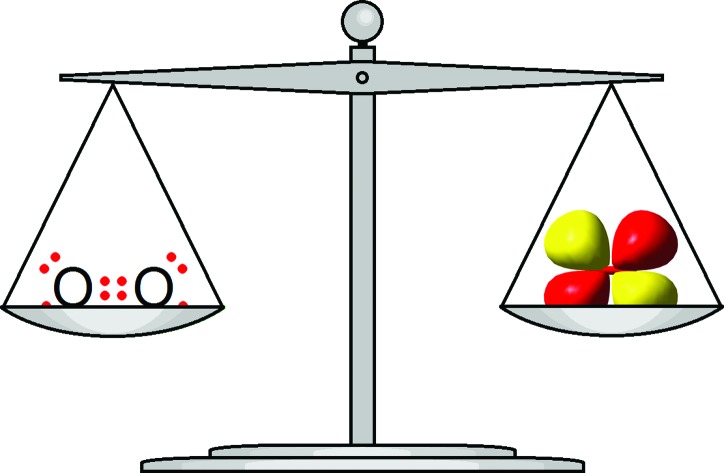
The Lewis and quantum mechanical theories of chemical bonding are compared and contrasted, with a view to clarifying the relationship between Harcourt’s ‘increased valence’ quantum approach and the recently proposed quantitative definition of hypervalency.



## 

In their comment and the associated references, Harcourt and Klapötke provide insights into the quantum descriptions of a variety of molecules, many of which can be considered as hypervalent by Musher's qualitative definition^1^ and also by the quantitative definition that I recently proposed.^2^ In particular, the ‘increased valence’ model developed by Harcourt^3^ clearly provides a valuable framework for the analysis of such molecules within the general context of valence bond (VB) theory. This much is not in dispute. Rather, the area of contention is the relationship between quantum calculations and the classical (*i.e.* non-quantum) Lewis model of chemical bonding.

It is worth restating some of the key differences between Lewis theory (LT) and quantum theory (QT). Thus, in LT, electrons are treated as classical particles that are individually localized in bonds or lone pairs, whereas in QT they show wave-particle duality and are delocalized into orbitals that may extend over several atoms. Similarly, in LT, bonds are formed by pairing electrons from neighbouring atoms such that all electrons are either bonding or non-bonding, whereas in QT electrons are located in orbitals that may be classified as bonding, non-bonding or antibonding. The importance of electron pairs in LT is undoubtedly connected to the fact that each electron in a molecule has a unique set of quantum numbers, of which the spin quantum number *S* can only take values of ±1/2. Nevertheless, the quantum mechanical concept of electron spin is wholly absent from the Lewis model. Conversely, the central importance that LT places on the octet rule for main group elements is not readily apparent from QT descriptions of molecules. Indeed, the double counting of electrons that is the essential step for obtaining such octets has no place in QT. As Gillespie has pointed out, this point has sometimes led to confusion when comparing LT and QT results.^4^

For the simplest molecule of all, namely H_2_, the LT picture of a two electron bond is in very good agreement with the QT picture of two electrons located in a 1σ_g_ bonding orbital, except of course that the LT model neglects the quantum property of electron spin pairing. For virtually all other molecules, the LT picture achieves conceptual simplicity at the expense of the deeper insights that are readily apparent from QT. To illustrate the point, consider the F_2_ molecule. According to LT, each atom in F_2_ achieves an octet of electrons by sharing a single bond with its neighbour, whilst retaining six unshared electrons in three lone pairs. In molecular orbital (MO) theory, all 14 of the valence electrons are in MO's that are delocalized over the whole molecule and the single bond arises from the difference in net allocations of electrons between bonding and antibonding orbitals. O_2_ provides an even more telling example. Here, the LT structure correctly predicts a double bond, but can offer no explanation for the molecule's paramagnetic ground state. QT not only confirms the double bond, but also provides a ready explanation for the paramagnetism in terms of the preferred electron spins in the ^3^Σ_g_^–^ ground state.

It is certainly true that a QT model can sometimes be overlaid on the more basic LT picture to provide additional insights; the archetype for this is provided by the Woodward–Hoffmann paradigm for pericyclic reactions. More generally, QT insights can often be used to extend the more elementary understanding of molecular electronic structure provided by LT. Indeed, Harcourt and Klapötke have used VB calculations for exactly this purpose in their comment. Nevertheless, there is an important difference between using QT to develop concepts that lie beyond the reach of LT, and using QT in an attempt to redefine LT. In particular, LT of itself has no conception of any bond apart from the **2c**–**2e** bond (albeit single, double *etc*.); consequently a few molecules, such as B_2_H_6_, cannot be described at all by classical LT, although it is easy enough to explain B_2_H_6_ in terms of the QT picture of **3c**–**2e** bonds. Similarly, the **2c**–**1e** bonds advocated by Harcourt and Klapötke are perfectly valid in QT, but are beyond the competence of LT. In the opinion of the author, it is better to use LT as received, albeit with a clear understanding of its shortcomings, than to attempt to hybridize LT with QT. To do otherwise is to risk compromising the conceptual simplicity of LT that is at the heart of its extraordinarily valuable insights.

Arguably the most natural interface between LT and QT is provided by Bader's atoms in molecules (AIM) theory. To quote Bader,^5^ ‘the possibility of obtaining a set of localized orbitals does not imply any physical localization of electrons … using the forms of individual orbitals to describe the charge distribution is both incorrect and ambiguous … these comments are, of course, not criticisms of the orbital model, which is *the* model for the prediction and understanding of the *electronic structure* of a many-electron system, but rather of the misuse of this model to describe the *spatial structure* of the electron distribution through illustrations of individual orbitals or their corresponding densities’ [emphasis in original]. This is precisely why the quantitative definition of hypervalency was developed using only the classical concept of atomic charges to extend the framework of Lewis' original model; any use of the QT concept of orbitals was carefully avoided.

Harcourt and Klapötke suggest that their VB models provide better insights into some molecules than the hypervalent Lewis structures given in ref. 2. This is as it should be; one would expect a QT model to provide deeper insights than the simpler LT model. Nevertheless, it is worth noting that the long, weak N–N single bond in N_2_O_4_ is also readily explained by the quantitative definition of hypervalency, since hypervalency is associated with instability and both of the N atoms involved in this bond are hypervalent. Concerning ozone, it is generally accepted that this molecule has substantial singlet diradical character. This is another important insight from QT and it should be mentioned that the analysis of O_3_ in ref. 2 pertained to the closed-shell singlet state. The equivalent unrestricted DFT calculation gives slightly different results [Δ*G* = +57.4 kcal mol^–1^; *γ*(O) = 9.64]. Nevertheless, the conventional Lewis picture of O_3_ can neither predict nor accommodate diradical character because LT does not include the concept of electron spin. It is also worth noting that individual resonance structures such as **3b** provided by Harcourt and Klapötke, although perfectly valid within VB theory, are invalid within LT as they do not observe the octet rule. In ref. 2, the accepted Lewis structure **5a** was taken as the starting point for the analysis of ozone.

Ultimately, the value of any model lies in its utility; specifically its power to explain or predict. In this regard, it is worth making the following observations. First, the quantitative relationship between *γ* values, as derived from AIM charges, and bond energies Δ*G*, provides a new and powerful validation of AIM charges compared to other charge schemes; this has been a point of considerable controversy in the literature.^6^ Second, that hypervalent molecules (as defined by *γ* values) are generally unstable is an important observation, which has until now been obscured by Musher's qualitative definition which includes many perfectly stable species such as phosphates. Indeed, we are currently investigating the role of hypervalent groups in explosives. Finally, the twin concepts of hypercoordination and hypervalency are no longer opposed, but rather complementary; molecules may be hypercoordinate but not hypervalent (*e.g.* SF_6_), hypervalent but not hypercoordinate (*e.g.* NNO), or both hypercoordinate and hypervalent (*e.g.* XeO_4_). When these concepts are combined with a few other simple ideas such as atomic radius and electronegativity, the Lewis model is seen to be more powerful than ever.
